# Tracking B cell immunity during perturbation of hepatitis B infection induced by treatment withdrawal

**DOI:** 10.1136/gutjnl-2024-333309

**Published:** 2026-07-08

**Authors:** Sabela Lens, Alice R. Burton, Jessica Davies, Maelle Locatelli, Mireia García-López, Anna Pocurull, Anna Jeffery-Smith, Nikolai Novikov, Simon P. Fletcher, Xavier Forns, Sofía Pérez-del-Pulgar, Mala K. Maini

**Affiliations:** 1 Institute of Immunity and Transplantation, Division of Infection and Immunity, UCL, London, UK; 2 Liver Unit, Hospital Clínic, IDIBAPS, CIBEREHD. University of Barcelona, Barcelona, Spain; 3 Gilead Sciences Inc., Foster City, CA, USA

**Keywords:** memory B cells, HBV functional cure, Nucleos(t)ide analogue withdrawal, hepatic flares, HBsAg loss

## Abstract

**Background:**

Withdrawal of prolonged nucleos(t)ide analogue (NA) treatment results in HBsAg loss in some subjects with chronic hepatitis B (CHB), potentially revealing immune correlates of functional cure.

**Objective:**

We investigated whether baseline or longitudinal changes in humoral immunity correlated with outcome of discontinuing prolonged NA treatment.

**Design:**

Global memory B cells (MBC) and T follicular helper cells (Tfh) were analysed by flow cytometry. HBs(small surface)/HBc(core)-MBC were quantified by *ex-vivo* bait staining and function assessed by cultured ELISpots. Immune parameters assessed at end-of-treatment (EOT), 12 and 48 weeks after treatment withdrawal (and at 4-8 years in a subset) were correlated with intrahepatic and longitudinal serum viral markers and ALT.

**Results:**

Individuals on prolonged NA had comparable frequencies of HBc-MBC and HBs-MBC, although the latter were PD-1^hi^ and functionally defective. Following treatment withdrawal, increases in class-switched HBc-MBC were frequently temporally linked with hepatic flares. Subjects achieving HBsAg loss had an increase in activated global MBC detectable at EOT that become more marked by week 48, accompanied by increases in plasmablasts. HBs-MBC in those with HBsAg loss showed significant reductions in PD-1, trends to increased activation (CD71) and function and a correlation with Tfh, compared to HBsAg persistence. MBC changes were maintained 4-8 years after HBsAg seroconversion.

**Conclusion:**

Differences in global and HBs-specific B cell immunity associate with HBsAg loss, whereas HBc-MBC temporally associate with flares, following withdrawal of prolonged NA treatment. Our results underscore the need to further explore the potential of B cell targets for monitoring and enhancing HBV functional cure in larger cohorts.

## Introduction

Understanding the interaction between hepatitis B virus (HBV) and the host immune response during chronic infection, as well as the impact of current nucleos(t)ide analogue (NA) therapy is crucial to inform novel treatment approaches aimed at functional cure. Interruption of NA therapy in the hepatitis B e antigen (HBeAg)-negative phase is one strategy that can achieve functional cure rates of up to 20% (1,2). Biomarkers are urgently required to more accurately select those who will benefit from NA withdrawal, which carries a risk of life-threatening ALT flares and viral rebound. Low serum quantitative hepatitis B surface antigen (qHBsAg) levels at the time of NA discontinuation identifies subjects with a higher probability of HBsAg loss(3,4), whilst serum hepatitis B core-related antigen (HBcrAg) and HBV-RNA have also been identified as useful predictors of viral control in some studies(5).

Viral markers reflect only part of the complex interplay between the virus and host immunity. Understanding the role of the immune system in mediating sustained virologic responses after NA withdrawal could identify additional biomarkers to predict effective NA withdrawal or reveal novel immunotherapeutic targets to increase the rate of HBsAg loss. Furthermore, NA withdrawal provides an opportunity to dissect immune correlates of HBsAg loss or sustained viral suppression that may shed light on immunopathogenesis and serve as biomarkers of functional cure in other settings. Our group and others previously described that the magnitude of HBV-specific T cell responses before or after NA cessation associates with the outcome of treatment interruption (4,6,7). However, none of these studies identified an association of T cell immunity with loss versus persistence of HBsAg. We therefore hypothesised that changes in B cells resulting from discontinuation following prolonged NA treatment may be a critical determinant distinguishing those subjects able to clear HBsAg and seroconvert.

Emerging data have revealed an unexpected role for B cell immunity in ongoing control of chronic hepatitis B (CHB), in addition to the well-established importance of neutralising antibodies in prevention of infection(8). HBV flares following therapies that deplete B cells have been extensively described in patients with both resolved and chronic infection, highlighting their relevance to ongoing control of residual HBV. *Ex vivo* identification of HBV-specific B cells using fluorescent bait reagents revealed that hepatitis B small surface (HBs)-specific memory B cells (MBC) persist in many individuals with CHB, despite lack of detectable anti-HBs Ab, although they were dysfunctional and present at lower frequencies than hepatitis B core (HBc)-MBC (9–11). Thus, HBs-specific B cells are numerically and functionally defective, despite their capacity to provide some ongoing contribution to HBV control, as previously described for T cells in chronic viral infections (12,13). Rescue of HBs-MBC function by PD-1 blockade combined with CD40L stimulation *in vitro* (9,10) raised the possibility that these populations might also be amenable to boosting *in vivo*. Critically, it is not known whether the emergence of detectable anti-HBsAb in cases of functional cure following NA withdrawal is simply a by-product of a reduction of HBsAg (resulting in less sequestration of bound HBsAb) or whether it also reflects some recovery in endogenous HBs-specific B cell function.

In this study we compared the kinetics of global, HBc and HBs-specific MBC frequencies and phenotype and activated circulating T follicular helper cells (cTfh) in HBeAg-negative CHB patients stopping NA therapy. We previously reported changes in HBV-specific T cells associating with sustained viral control but not distinguishing those with loss or persistence of HBsAg in this cohort (4). The availability of five longitudinally sampled individuals who achieved HBsAg loss provided a unique opportunity to determine whether their functional cure was associated with any differences in B cell phenotype and function compared to the rest of the cohort.

## Methods

### Patients

This was a prospective single-centre study involving 21 patients with HBeAg-negative CHB with complete virological control (undetectable HBV-DNA and normal ALT levels) for at least 3 years under NA therapy. Samples used for this study represent the subset of the previously published cohort (4) from whom sufficient PBMC remained. Exclusion criteria included advanced liver disease (F3–F4 according to METAVIR by liver biopsy or previous diagnosis of cirrhosis), immunosuppressive therapy, hepatocellular carcinoma, or coinfection with HIV, HCV, or HDV.

### Ethics statement

The study was approved by the Hospital Clinic and Brighton and Sussex Ethics Committee (references 2015-0453/2018-0370 and 11/LO/0421; respectively) and was conducted in compliance with the principles of the Declaration of Helsinki, Good Clinical Practice guidelines, and local regulatory requirements. All patients provided written informed consent before inclusion.

### Study design

**Fig. 1:**
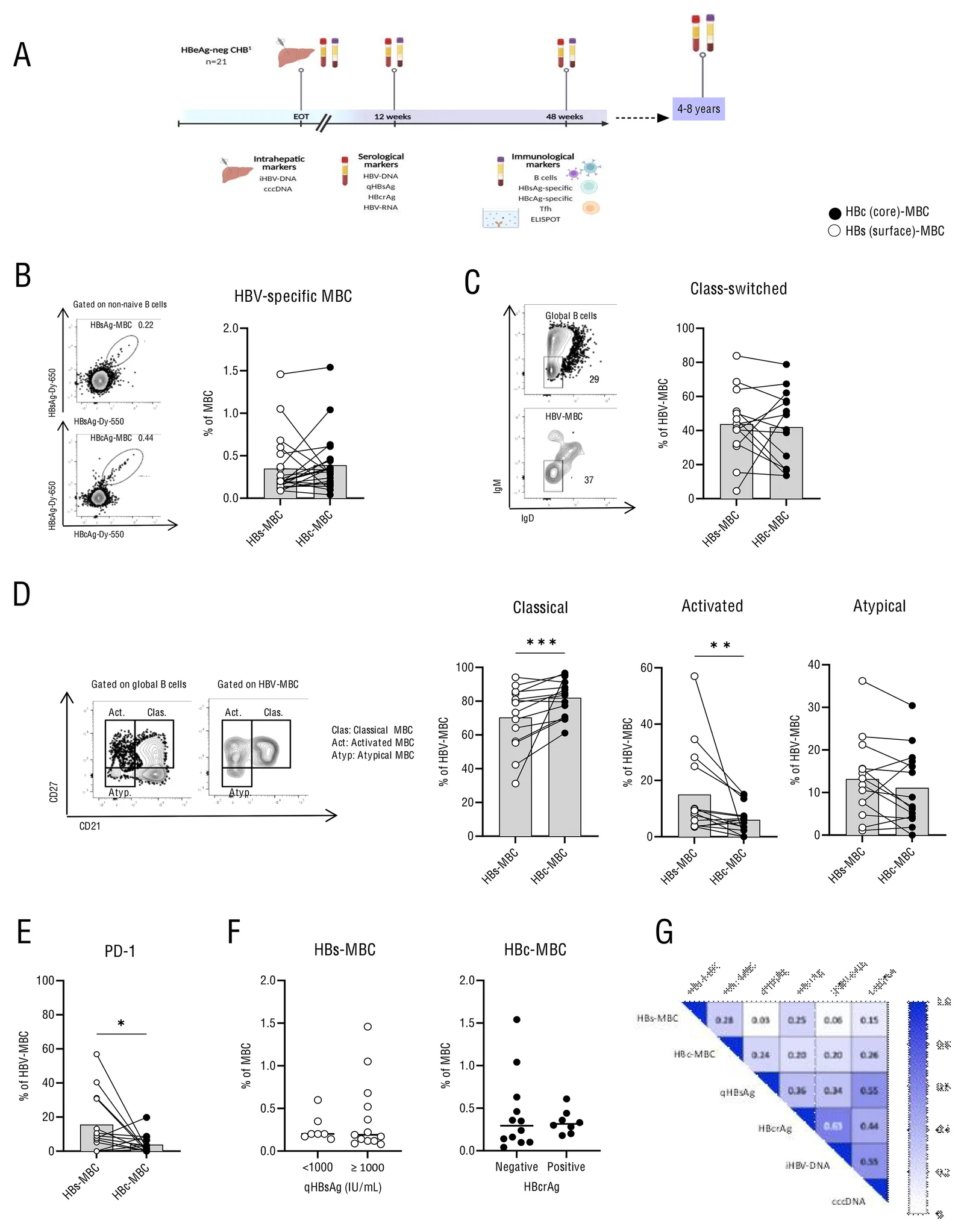
Comparable frequencies of total and class-switched HBs and HBc-MBC following prolonged NAs. (A) Study design. (B) Representative FACs plot and paired frequencies of HBc and HBs-MBC and (C) of class-switched cells within HBV-MBC. (D) Representative FACS plot and frequencies of MBC subsets within HBc and HBs-MBC. (E) Paired comparison of PD-1+ expression on HBV-MBC. (F) Frequency of HBs-MBC according to qHBsAg levels and frequency of HBc-MBC according to HBcrAg detectability. (G) Heat map showing correlations between serum, intrahepatic viral markers and HBV-MBC frequencies. Open dots represent HBs-MBC and closed dots represent HBc-MBS. Wilcoxon’s paired t test (B-E); Mann-Whitney test (F); Spearman correlation (H). *p<0.05; **p<0.01.

Patients underwent a liver biopsy before treatment withdrawal (end of treatment, EOT). Serum samples and peripheral blood mononuclear cells (PBMCs) were obtained at baseline, 12, and 48 weeks after NA discontinuation and, in a subset of 7 patients, a last FU sample was acquired 5 (4–8) years after NA discontinuation. Assessments after treatment interruption and re-treatment criteria have been previously specified (4). Sampling time-points and analysis are summarized in Fig.1a. In addition, 6 vaccinated healthy controls with detectable HBsAb (>10 IU/mL) were used for ELISpot comparison.

### B cells and circulating Tfh (cTfh)

PBMCs were isolated from heparinized blood by density centrifugation using Ficoll-Hypaque Plus (GE Healthcare). PBMCs were frozen in FBS (Life Technologies) supplemented with 10% DMS0 (Sigma-Aldrich). 16-colour flow cytometry was used for ex vivo analysis of B cells and cTfh. Full details of mAbs used, including those used for phenotypic characterization, are given in Suppl. Table 1. Cells were stained with Fixable Live/Dead Dye (Life Technologies, Thermo Fisher Scientific) before incubation with saturating concentrations of surface mAbs diluted in 50% Brilliant Violet Buffer (BD Biosciences) and 50% PBS for 30 minutes at 4°C. In all instances, cells were stained in the presence of FcR-blocking reagent (Miltenyi Biotec). All samples were acquired on a Fortessa-X20 (BD Biosciences) and analyzed using FlowJo (v10).

### HBV-specific memory B cells (MBC)

For identification of antigen-specific B cells, 1 × 10^6^ cells were stained with two fluorescently conjugated bait reagents with either HBs (small S antigen) or HBc (DyLight550 and DyLight650, provided by Gilead Sciences). HBc-D550 and - D650 were used at 600 ng/ml; HBs-D550 and -D650 at 20µg/ml. All staining was performed in parallel with mAb staining and diluted in 50:50 Brilliant Stain Buffer (BD Biosciences) and PBS. Stringent gating criteria were applied with doublet, dead, and CD19-negative cell exclusion to minimize nonspecific binding contamination. Cells were stained with an identical panel minus baits (fluorescence minus one [FMO]) to optimize the gating. The phenotype of antigen-specific memory B cells was not analysed in instances in which there were fewer than 20 double-bait positive cells recorded.

### Virological serum and intrahepatic markers

see Supplementary

### Elispot

To promote B cell differentiation into Ab-secreting cells, PBMC were first incubated with 1μg/ml R848 and 20 IU/ml IL-2 in cRPMI for 5 days in 24-well plates. Multiscreen HTS-IP (Merck Millipore) plates were coated with 1μg/ml HBsAg, 0.5 μg/ml HBcAg (kind gift from Dynavax) or 1 μg/ml anti-human IgG overnight at 4oC. Plates were then washed with PBS and blocked with RPMI + 10% FBS for 2h at 37°C. Prior to seeding, cells were washed and resuspended in cRPMI. The number of cells seeded varied according to the antigen of interest: a total of 5 × 10^5^ (HBsAg), 1 × 10^4^ (HBcAg) and 1 × 10^3^ (IgG) PBMCs were plated per well and incubated at 4°C for 24 hours. Cells were removed and wells washed with filtered PBS + 0.05% Tween 20 (PBS–T) followed by filtered PBS. Goat anti-human IgG-horseradish peroxidase (anti-IgG-HRP, Jackson Immuno Research Laboratories; 1μg/ml in PBS + 10% FBS) was added and incubated for 4 hours at RT in the dark. Wells were again washed 3 times with filtered PBS–T and 3 times with filtered PBS and then developed with AEC substrate (BD Biosciences), according to the manufacturer’s instructions. All assays were performed in triplicate. Spot-forming units (SFU) were imaged and counted using an AID ViruSpot reader.

### Statistics

Statistical analyses were performed in Prism (GraphPad version 10) as indicated in legends: Kruskal-Wallis test (ANOVA) with Dunn’s post hoc test for pairwise multiple comparisons, Spearman’s rank correlation, Mann-Whitney unpaired t test and Wilcoxon’s paired t test. All tests were carried out as 2-tailed tests. Significance was defined as p < 0.05.

### Patient and Public involvement

Patients and members of the public were not involved in the design, conduct, reporting, or dissemination plans of this research.

## Results

### Comparable frequencies of class-switched HBs and HBc-specific MBC following prolonged NA treatment

To examine the dynamic changes in B cell immunity during differential outcomes of CHB we took advantage of a cohort of HBeAg-negative subjects undergoing NA withdrawal after long-term antiviral treatment (Fig.1a). In addition to thorough longitudinal blood monitoring, this cohort had a liver biopsy at the time of treatment withdrawal, allowing assessment of intrahepatic HBV replication. Cryopreserved PBMC were available from a subset of the original cohort (n=21 of total 27) in whom we previously reported virus-specific T cell immunity (4). The overall demographics and baseline clinical characteristics of the available study cohort are shown in Suppl.Table 2; most were Caucasian and 81% male, with a median age of 57 years. Median NA therapy duration was 8 years, with a minimum of 3 years; 16 (76%) had received tenofovir and 5 (24%) entecavir.

We first compared the baseline profile of HBc and HBs-specific MBC at the EOT, just before NA withdrawal. To assess their circulating frequencies and phenotype/isotype we used multiparameter flow cytometry following direct *ex vivo* staining (gating strategy Suppl.Fig.1a), with dual-fluorochrome-labelled antigen baits whose functional specificity has been previously confirmed (10,11,14). Previous studies of untreated CHB have reported frequencies of HBc-specific B cell frequencies that are 5-18 fold higher than those of HBs-specific B cells (11,14). By contrast, in our long-term NA suppressed cohort, there was no significant difference in HBc and HBs-specific B cell frequencies (Fig.1b). The proportion of class-switched MBC after prolonged NA suppression was also equivalent within the two specificities (Fig.1c), whereas these were previously found to be markedly reduced in HBs-MBC compared to HBc-MBC in untreated CHB (11). The enrichment of atypical (CD27^-^CD21^-^) MBC that is characteristic of responses directed against HBs compared to HBc in untreated CHB (11) was also not significant in our NA-treated cohort, although some individuals did show an enrichment of activated (CD27^+^CD21^-^) HBs-MBC with a corresponding decrease in classical (CD27^+^CD21^+^) HBs-MBC (Fig.1d). Moreover, PD-1 expression was increased on HBs-MBC compared to HBc-MBC (Fig.1e). Thus, at the time-point just prior to withdrawal of prolonged NA, HBs-MBC showed comparable frequencies and class-switching to HBc-MBC but were enriched for activated MBC and PD-1 expression.

We next looked at the inter-individual variations in HBV specific B cell frequencies at the time of NA withdrawal to determine if these associated with any circulating or intrahepatic viral markers. Peripheral viral markers did not correlate with frequencies of antigen-specific MBC. Of note, there was no correlation between circulating HBs-MBC frequencies and serum concentrations of this viral antigen after prolonged NA treatment (Fig.1f and Suppl.Fig.1b), as previously reported in untreated CHB (9,10). Similarly, the detection or concentration of HBcrAg did not associate with frequencies of HBc-MBC (Fig.1f and Suppl.Fig.1c). Taking advantage of our access to compartmentalised liver viral markers, we compared cccDNA and total intrahepatic HBV-DNA levels with peripheral viral markers and B cell immunity. A correlation matrix showed the previously reported significant correlation of serum HBsAg with intrahepatic cccDNA and of serum HBcrAg with intrahepatic HBV-DNA in this cohort (4) (Fig.1g). However, baseline HBs and HBc-MBC frequencies did not correlate with any measures of intrahepatic HBV. In summary, the variability between individuals in antigen-specific B cell profiles before interruption of prolonged NA does not associate with peripheral or intrahepatic viral parameters.

### Baseline B cell profiles as predictors of treatment interruption outcome

**Fig. 2:**
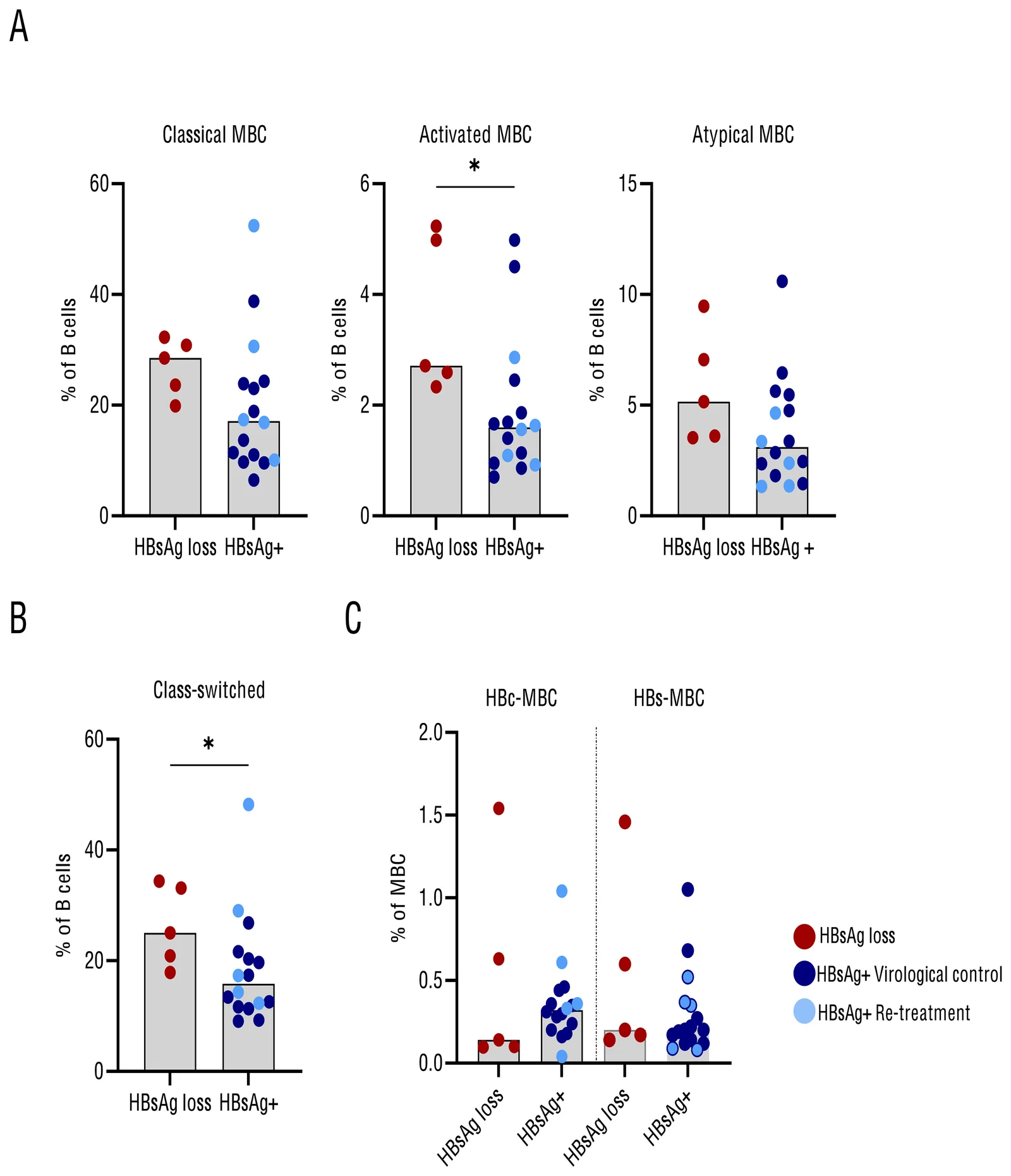
B cell profiles at EOT according to HBsAg clinical outcome after NA discontinuation. (A) Individual frequencies of MBC subsets at EOT. (B) Frequency of global class-switched B cells. (C) Frequency of HBc and HBs-MBC cells. Red dots represent patients with HBsAg loss. Blue dots represent patients with persistent HBsAg either with off-treatment sustained viral control (dark blue) or re-treatment (light blue). Bars indicate median. Mann-Whitney test (A-C). * p<0.05.

Following treatment interruption in this cohort, 5 individuals (24% of the total cohort) achieved functional cure with loss of HBsAg and anti-HBs antibody seroconversion, whilst the remaining 16 maintained detectable HBsAg. Of those remaining HBsAg positive, 11 patients (52%) achieved viral control not requiring re-treatment and 5 needed re-treatment after both an ALT flare and DNA rebound (Supp.Fig2a,b). As previously reported for the full cohort (4), EOT serum HBsAg, intrahepatic HBV-DNA and cccDNA levels were lower in individuals who went on to achieve HBsAg loss (Supp.Fig.2c).

We next examined whether global and antigen-specific B cell profiles at the time of treatment interruption were predictive of virological outcome, dividing the cohort into those with or without subsequent HBsAg loss (red and blue dots respectively). The memory phenotype of global B cells differed at EOT according to the ensuing HBsAg outcome, with activated (CD27^+^CD21^-^) and class-switched (IgD^-^IgM^-^) B cells (considered indicative of ongoing antiviral responses (15,16)), already being significantly, although subtly, enriched in those going on to achieve HBsAg loss versus persistence (Fig.2a,b). Other memory subsets showed non-significant trends to be enriched in the group with HBsAg loss (Fig.2a). Global B cell subsets did not differ between the two groups with HBsAg persistence with or without requirement for re-treatment (light and dark blue dots respectively, Fig.2a,b). Frequencies of HBs and HBc-MBC (Fig.2c) (and memory phenotype in samples with sufficient of these small populations to assess, Suppl.Fig.2d) also showed no significant differences according to HBsAg loss or persistence. Other global B cell phenotypic markers (CXCR5, CD71, CD40, indicative of lymph node homing, activation and T cell interactions, respectively) showed no differences when stratifying according to clinical outcome of treatment interruption in either the global or virus-specific B cell compartment (Supp.Fig.2e and 2f).

Thus data from this cohort raised the possibility that low frequencies of class-switched and activated MBC after prolonged NA may be predictive of HBsAg persistence in the treatment withdrawal setting.

### Increases in class-switched HBc-specific MBC cells following treatment withdrawal

**Fig. 3:**
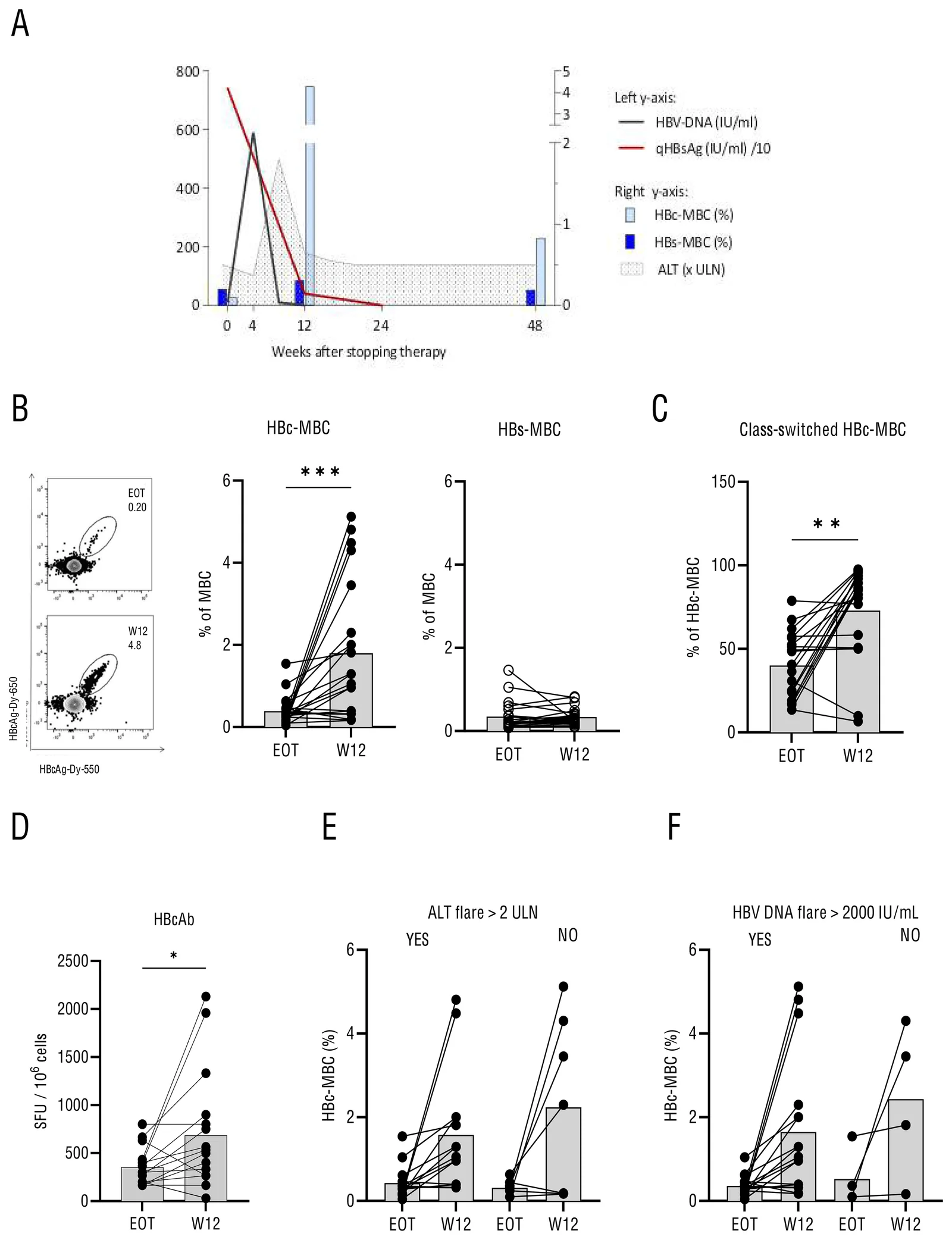
Dynamic changes in HBc- and HBs-MBC after treatment withdrawal. Representative example of HBV-DNA, qHBsAg, ALT and HBV-MBC kinetics after treatment discontinuation in a patient achieving HBsAg loss. (B) Representative FACS plot of HBc-MBC change and paired frequencies of HBc and HBs-MBC at EOT and W12. (C) Increase in class-switched HBc-MBC and (D) HBcAb production by ELISpot at W12. (E) Dynamics of HBc-MBC at EOT and W12 according to ALT or (F) HBV-DNA flares. Bars indicate median. Wilcoxon’s paired t test (B-F); * p<0.05; **p<0.01; ***p<0.001.

The frequency of B cells specific for HBc (core) has previously been noted to be lower in individuals on antiviral therapy or with resolved infection compared to untreated CHB (11,14). Conversely, HBc-MBC were increased cross-sectionally in CHB stages with raised ALT and also fluctuated longitudinally in 4 patients sampled during and/or after spontaneous hepatic flares (11,14). We therefore hypothesised that the frequency of HBc-MBC may increase during the viral or hepatic flares often seen after stopping NA. Fig.3a shows the prototypic temporal dynamics following NA withdrawal in a representative patient achieving HBsAg loss 24 weeks after stopping NA; rebound in viral DNA peaked at week 4, followed by an ALT flare peaking at week 8, with HBc-MBC increasing ~8-fold by the first sample after withdrawal (week 12) and declining towards baseline values by week 48, contrasting with stable frequencies of HBs-MBC throughout. The frequency of HBc-MBC increased in the majority of subjects when sampled 12 weeks after stopping NA compared to the end of treatment time-point, whereas those directed against HBs did not (Fig.3b). Characterisation of the expanded pool of HBc-MBC revealed an increase in the proportion that was class-switched at week 12, whilst atypical MBC decreased and the remaining MBC subsets remained stable (Fig.3c, Suppl Fig.3a). In parallel, HBc-MBC functionality assessed by ELISpot also showed an increase in the HBcAb production at week 12 (Fig.3d).

The increase in the frequency of HBc-MBC following treatment discontinuation was not consistently linked with any one clinical parameter. There was a mean 4-fold increase in HBc-MBC from EOT to week 12, with responses increasing from <1% to >4% of the overall memory B cell pool in 4 subjects and increasing to some degree in 12 out of the remaining 17. Although an increase in HBc-MBC was more frequent in subjects with an ALT flare (>2x upper limit of normal), there were clear exceptions, with some showing an increase in HBc-MBC with no detectable flare and others showing no change in the frequency of HBc-MBC at week 12, despite a marked ALT flare (Fig.3e). The week 12 increase in HBc-MBC was also not consistently accompanied by an increase in HBV DNA or HBcrAg (Fig.3f, Suppl.Fig.3b). By week 48, when clinical parameters had stabilised, HBc-MBC frequencies had reduced back down in most subjects regardless the clinical outcome (Suppl.Fig.3c).

In summary, frequencies and class-switching of HBc-MBC increased in most individuals after treatment withdrawal, often, but not uniformly, associating with their withdrawal flare.

### Signatures of reduced B cell dysfunction coordinated with Tfh activation associate with HBsAg loss

**Fig. 4:**
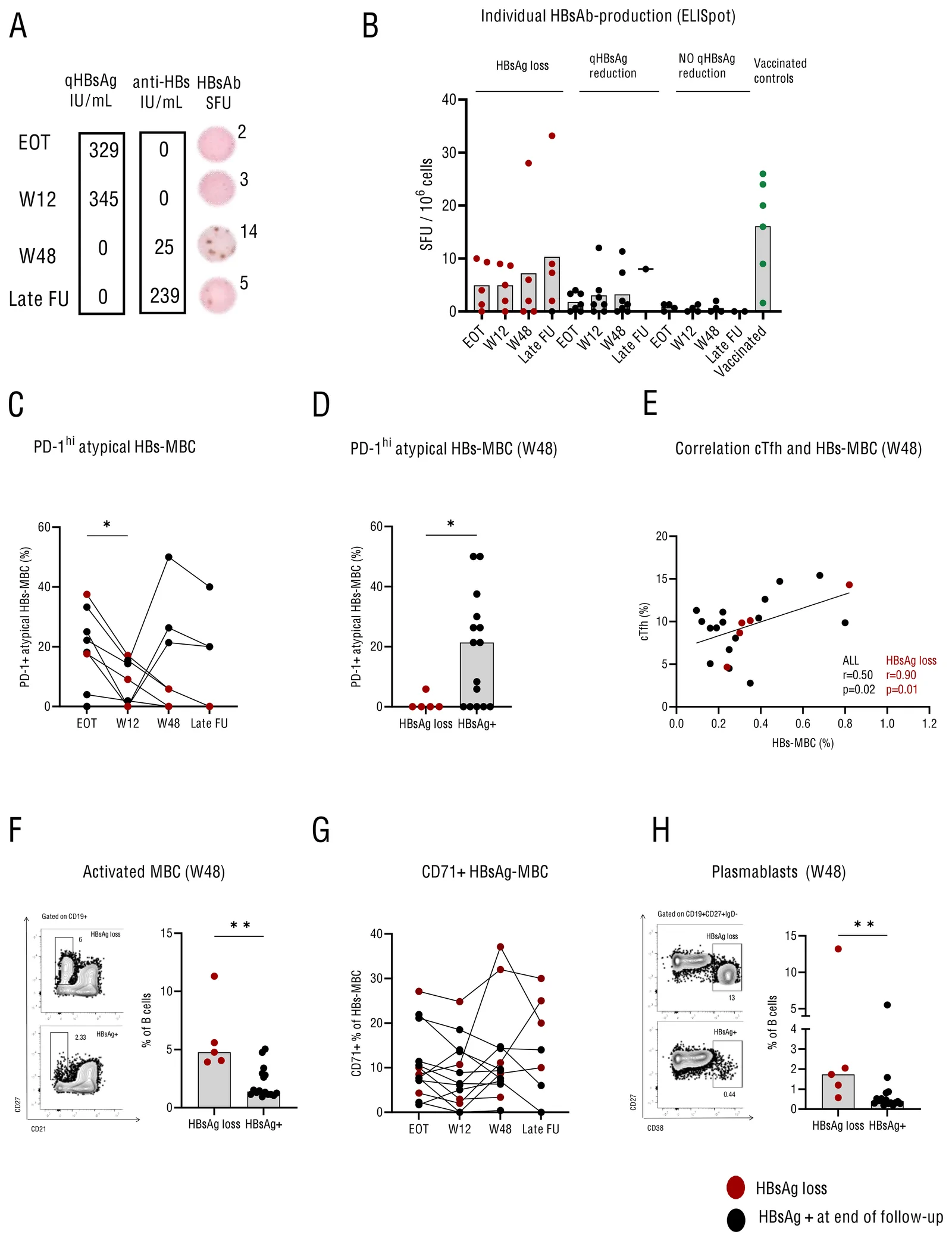
Signatures of reduced B cell dysfunction coordinated with cTfh activation associate with HBsAg loss. (A) representative example of HBsAb production by ELISpot in parallel with qHBsAg and anti-HBs-Ab levels in serum (B) Longitudinal HBsAb production assessed by ELISpot (SFU/10^6^ cells) according to qHBsAg outcomes: HBsAg loss (n=5), qHBsAg reduction (n=7) or not (n=4) (average from triplicates) compared to non-infected HBV-vaccinated controls. (C) PD-1 expression on atMBC within HBs-MBC based on HBsAg status longitudinally and (D) at W48 only in patients with sufficient atypical HBs-MBC cell count at all time points (n=11). (E) Correlation between cTfh and HBs-MBC at W48. (F) Activated MBC frequencies according to HBsAg status at W48 and representative FACS plot. (G) Frequency of CD71^+^HBs-MBC after NA discontinuation according to HBsAg outcome among those with sufficient HBs-MBC cell count (n=13) (H) Frequency of Plasmablasts at W48 according to HBsAg status and representative FACs plot. Bars indicate median. Red and black dots represent patients with HBsAg loss or not; respectively and green vaccinated controls. Mann-Whitney test (D,F,H); ANOVA or Friedman test with multiple comparisons (B,C,G); Spearman correlation (E) * p<0.05; **p<0.01

The optimal outcome of treatment interruption and other approaches to functional cure is HBsAg loss with anti-HBs antibody seroconversion. Five of this cohort lost HBsAg; late FU in this subset demonstrated that all of these eventually seroconverted (anti-HBs levels between 20 and 1000 IU/mL by 4 to 8 years, Suppl. Table 3). Serology remained negative for anti-HBsAb at every time point for the other subjects with HBsAg persistence. We next focused on detecting any differences in humoral immunity that might be contributing to, or resulting from, this change in HBsAg load after treatment withdrawal. Frequencies of HBs-MBC at week 48 did not differ between the group with or without HBsAg loss (Suppl.Fig.4a). However, previous studies have shown that although HBs-MBC can often be detected in CHB, they are highly dysfunctional, with impaired capacity to differentiate into antibody-secreting cells (9,10). Therefore, it was important to functionally assess the ability of these cells to differentiate in vitro to produce anti-HBs antibodies, measured by cultured ELISpot (spot-forming units –SFU-per 10^6^ cells). We compared functional responses of HBs-MBC at different time points after NA discontinuation in 16 patients with available samples, grouped by changes in circulating quantitative HBsAg: HBsAg loss, HBsAg reduction (at least 1 log IU/mL reduction from baseline), and no HBsAg reduction. To assess durability, we obtained late FU PBMC 4 to 8 years after treatment withdrawal from 7 patients (4 of those losing HBsAg and 3 with HBsAg persistence). Individuals who achieved HBsAg loss tended to show functional potential to develop anti-HBs Ab responses, with a subtle trend to increase over time, such that all 4 sampled at late FU 4-8 years after NA withdrawal had positive cultured ELISpots, (representative example, Fig.4a). In contrast, those with no reduction in qHBsAg had consistently negligible or undetectable functional responses, whilst those with partial HBsAg reduction showed intermediate responses (Fig.4b). Thus, memory B cells had the potential to differentiate into anti-HBs Ab-producing plasmablasts in vitro in subjects achieving HBsAg loss, that resembled responses from vaccinated controls and differed from those with HBsAg persistence (Fig.4b). However, the antibody-producing capacity of HBs-MBC, as assessed by cultured ELISpots, remained markedly lower than that of HBc-MBC and showed no correlation within individuals (Suppl.Fig.4b and c). Moreover, unlike HBs ELISpots, HBc ELISpots were comparable in all 3 groups of patients when divided by HBsAg loss or persistence (Suppl.Fig.4d).

We and others previously showed that functional recovery of HBs-MBC from patients with CHB could be achieved *in vitro* by reducing signals to PD-1^hi^ atypical MBC (using anti-PD-1 blocking antibodies) and enhancing CD40 stimulation to mimic T follicular cell help (9,10). The raised expression of PD-1 on atypical HBs-MBC decreased significantly at week 12 after treatment withdrawal (Fig.4c), irrespective of whether there was any HBsAg decrease by this timepoint (Suppl.Fig.2b) (and without any change in atypical MBC frequency, Suppl.Fig.4e). However, PD-1 levels on atypical HBs-MBC rebounded back up by week 48 in those with HBsAg persistence (Fig.4c). Therefore, cross-sectional comparison at week 48 showed a significant reduction in PD-1 levels on atypical HBs-MBC in the group with HBsAg loss compared to those with HBsAg persistence (Fig.4d). In patients who achieved HBsAg loss, PD-1 expression on atypical HBs-MBC became negligible by week 48 and remained low at late FU (Fig.4c). As a proxy measure for the activity of Tfh sequestered in lymph node germinal centres, we then quantitated the frequency of CD38^+^ activated Tfh detectable in the circulation (cTfh) (17). The proportion of cTfh expressing CD38 increased consistently at week 48 after NA withdrawal, including in the HBsAg loss group (Suppl.Fig.4f). Notably, the frequency of cTfh correlated significantly with that of HBs-MBC and this correlation was particularly strong (r=0.9) in the subset of subjects achieving HBsAg loss (Fig.4e).

By week 48 there was maintenance and/or further increases in the activated fraction of MBC that we had already noted to be enriched at EOT (Fig.2a), selectively in the HBsAg loss group (Fig.4f, Suppl.Fig.4g). In parallel, the HBs-MBC compartment showed an increase in the activation marker CD71 in patients achieving HBsAg loss (Fig.4g). In subjects achieving HBsAg loss there was also an increase in plasmablasts (marked by high co-expression of CD38 and CD27) such that they were also significantly enriched at week 48, compared to those with HBsAg persistence (Fig.4h, Suppl.Fig.4h). After 4-8 years, activated MBC (CD27+CD21-B cells and CD71+ HBs-MBC) persisted in HBsAg seroconverters, whereas, as expected, circulating short-lived plasmablasts decreased (Fig.4f-h and Suppl Fig.4f-h).

Thus, changes triggered by NA withdrawal in the HBsAg loss group included a reduction of PD-1 expression on atypical HBs-MBC coordinated with an increase in activated cTfh, conditions favouring memory B cell activation and plasmablast development for the production of anti-HBs antibodies.

## Discussion

Unravelling the immune correlates of viral control and liver damage is complex in the steady-state setting of a long-established infection like CHB. However, treatment interruption provides a window to assess longitudinal immunological changes accompanying the resultant dynamic disease fluctuations within individuals. Further insights may be gained by comparing immune profiles across groups with contrasting outcomes of this intervention. Using this approach, previous studies have identified more functional HBV-specific T cells associating with lack of flares or sustained viral control following treatment withdrawal, but this arm of the immune response did not correlate with the loss of HBsAg [4,6,7]. Recent data indicate that functional recovery of HBs-specific B cells can occur in some patients who achieve functional cure on Peg-IFNα treatment (19). To our knowledge, this is the first study to investigate HBV-specific B cell responses in the context of NA treatment interruption. While our findings point to potential immunological signatures that could be linked with HBsAg seroconversion, the small number of cases precludes definitive conclusions, and these observations should be considered exploratory.

There are striking differences in the humoral response to the capsid (HBc) and envelope (HBs) of HBV. HBcAg elicits strong T cell independent and dependent antibody responses (20), typically sustained for decades regardless of whether HBV is resolved or chronic, whereas antibodies to HBsAg are only detectable following natural or treatment-induced resolution of infection. Recent studies showed that HBs-MBC do actually persist in most individuals with CHB but are lower frequency, more enriched for an atypical PD-1^hi^ phenotype and less functional than their HBc-MBC counterparts. In this study we observed that individuals recruited for treatment interruption, who had been taking NA for a median of eight years, had lost some of these features distinguishing their HBs- and HBc-MBC. The equalisation of MBC frequencies of these different HBV specificities was likely partly attributable to decreases in HBc-MBC induced by NA, as previously described (14). However, the possibility that long-term treatment had also induced partial restoration of HBs-MBC was suggested by their frequencies being higher than those reported in untreated CHB using the same reagents (11,14), together with their changes in memory phenotype and class-switching. This latter possibility would need confirmation by longitudinal study of individuals starting NA.

Although HBs-MBC frequencies after long-term NA therapy were comparable with those of HBc-MBC, their antibody-producing capacity (assessed by cultured ELISpots) remained markedly lower, consistent with previous findings in untreated CHB patients (11,14). The impaired function of HBs-specific B cells was previously shown to be amenable to rescue *in vitro* through a combination of PD-1 blockade and Tfh signals (CD40L and IL-21) (9,10). Our findings in individuals losing HBsAg in this study collectively support some functional recovery of their humoral response *in vivo* also being linked to a change in the balance of PD-1 and Tfh signals following NA withdrawal. Although a sustained reduction in PD-1 on HBs-MBC associated both temporally and cross-sectionally with seroclearance of HBsAg, it was not possible to distinguish whether this was cause or effect. It will be instructive to examine the impact on HBs-MBC functionality achieved by other novel therapeutic approaches able to directly and rapidly lower HBsAg, such as monoclonal antibodies and siRNA. Current trials of *in vivo* PD-1 blockade should also examine whether this is sufficient to reconstitute Tfh help and rescue some HBs-specific B cell immunity.

Our study was limited in its capacity to robustly identify B cell correlates of functional cure by the small number of cases with HBsAg loss and seroconversion. In addition, memory B cells, cTfh and plasmablasts are predominantly sequestered in lymphoid tissue, whereas we relied on changes detectable in blood. In particular, low circulating frequencies and limited sample availability restricted the comprehensive characterization of cTfh and antigen-specific B cells in our study. Despite these caveats, we were able to detect signatures of enhanced B cell immunity, with functional HBs-MBC expressing less PD-1, activated cTfh, activated memory B cells, and plasmablasts associating with HBsAg loss. Ideally, a novel immunological biomarker for functional cure would be detectable at the EOT time point, in order to avoid unnecessary risks of detrimental side effects in those less likely to respond favourably to NA withdrawal. Data from our small cohort suggested that differences in activated memory cells within the global B cell compartment may already be detectable at EOT according to subsequent HBsAg loss. This could be adapted for quantitation in larger clinical trials by a simple flow cytometric panel not requiring a specialist immunology laboratory (analogous to CD4 counts for management of people living with HIV). However we acknowledge that the small sample size and single-center design limit the generalizability of our findings. Future larger, multicenter studies will be essential to validate these immune correlates of functional cure and assess their potential utility as biomarkers in broader clinical settings.

An additional striking observation in this cohort was the consistent increase in frequencies of class-switched HBc-MBC following treatment withdrawal, not reflected in the HBs-MBC compartment. This capacity of HBc-MBC to be boosted rather than tolerised by increases in their cognate antigen may relate to the highly immunogenic properties of the icosahedral HBcAg, including its lack of reliance on T cell help. A recent study detected naked capsids (containing HBV-RNA and other replicative intermediates), circulating as higher-order antigen-antibody complexes with anti-HBc Ab in CHB (21); these would be expected to potently activate complement, providing a putative mechanism for the increase in HBc-MBC to contribute to liver inflammation. Alternatively, liver damage initiated by other pathways could increase the release of HBcAg from damaged hepatocytes and drive expansion of HBc-MBC. The increase in class-switched HBc-MBC was detectable at week 12 and declined by week 48; more detailed temporal analysis would be needed to see if it peaked with viral rebound or the subsequent ALT flare, as reported in 4 previous cases of HBV flares (11). The fact that increases in HBc-MBC were seen in some individuals not experiencing with ALT flares or detectable increases in HBcrAg may reflect that these factors are not inextricably linked or that clinical assays are not sensitive enough to detect, for example, increases in capsid immune complexes. By contrast, HBs-specific B cells did not display a comparable magnitude of change, underscoring their impaired responsiveness to antigenic fluctuations.

In summary, prolonged NA can equalise frequencies but not PD-1 status or function of class-switched MBC directed against HBc and HBs. Our results provide further evidence that chronic HBV infection is associated with an intrinsic defect in HBs-specific B cell function, rather than the lack of anti-HBsAb being solely attributable to antigen sequestration. Although B cell parameters show no association with either peripheral or intrahepatic viral markers in the EOT steady state, the rapid viral changes induced by NA withdrawal are associated with distinct changes in B cell immunity. The marked increases in HBc-MBC following NA withdrawal underscore their potential to sensitively reflect disease activity and the need for further study to understand potential mechanistic links. The combined signatures suggesting some enhanced humoral immunity associating with HBsAg loss upon NA withdrawal raise the prospect of achieving more complete recovery of endogenous B cell function with more targeted immunotherapeutic approaches. Studies in larger cohorts will also be needed to assess the potential utility of activated MBC or other B cell biomarkers to add to the ability of HBsAg levels to discriminate patients with a higher chance of HBsAg loss after NA interruption.

## Supplementary Material

Supplementary information

## What is already known on this topic

- Functionally defective, atypical, PD-1^hi^ HBs-specific memory B cells (MBC) persist in untreated CHB at lower frequencies than HBc-MBC; it is not known if these differences remain after prolonged NA therapy.
- HBsAg seroconversion occurs in up to 20% of patients following NA withdrawal in HBeAg-negative CHB, but it has not been investigated whether this is driven by, or results in, any changes in HBs-MBC.

## What this study adds

- Prolonged NA therapy equalises frequencies of class-switched MBC specific for HBc and HBs but PD-1 expression of HBs-MBC is only reduced following treatment withdrawal, with sustained reductions restricted to those undergoing HBsAg loss.
- NA withdrawal outcome is reflected in other aspects of B cell immunity including higher activated MBC, plasmablasts and HBs-MBC functional potential in those with HBsAg loss, whereas increases in class-switched HBc-MBC associate temporally with withdrawal flares.

## How this study might affect research, practice or policy

- Results highlight the need for larger studies exploring the utility of B cell biomarkers to predict responders in the setting of NA therapy discontinuation and to guide new therapeutic targets to enhance functional cure rates.

## References

[1] Lampertico P, Agarwal K, Berg T, Buti M, Janssen HLA, Papatheodoridis G (2017). EASL 2017 Clinical Practice Guidelines on the management of hepatitis B virus infection. J Hepatol.

[2] Berg T, Simon KG, Mauss S, Schott E, Heyne R, Klass DM (2017). Long-term response after stopping tenofovir disoproxil fumarate in non-cirrhotic HBeAg-negative patients - FINITE study. J Hepatol.

[3] Hirode G, Choi HSJ, Chen CH, Su TH, Seto WK, Van Hees S (2022). Off-Therapy Response After Nucleos(t)ide Analogue Withdrawal in Patients With Chronic Hepatitis B: An International, Multicenter, Multiethnic Cohort (RETRACT-B Study). Gastroenterology.

[4] García-López M, Lens S, Pallett LJ, Testoni B, Rodríguez-Tajes S, Mariño Z (2021). Viral and immune factors associated with successful treatment withdrawal in HBeAg-negative chronic hepatitis B patients. J Hepatol.

[5] Carey I, Gersch J, Wang B, Moigboi C, Kuhns M, Cloherty G (2020). Pregenomic HBV RNA and Hepatitis B Core-Related Antigen Predict Outcomes in Hepatitis B e Antigen-Negative Chronic Hepatitis B Patients Suppressed on Nucleos(T)ide Analogue Therapy. Hepatology.

[6] Rivino L, Le Bert N, Gill US, Kunasegaran K, Cheng Y, Tan DZM (2018). Hepatitis B virus – specific T cells associate with viral control upon nucleos (t) ide-analogue therapy discontinuation.

[7] Rinker F, Zimmer CL, Höner zu Siederdissen C, Manns MP, Kraft ARM, Wedemeyer H (2018). Hepatitis B virus-specific T cell responses after stopping nucleos(t)ide analogue therapy in HBeAg-negative chronic hepatitis B. J Hepatol.

[8] Maini MK, Pallett LJ (2018). Defective T-cell immunity in hepatitis B virus infection: why therapeutic vaccination needs a helping hand. Lancet Gastroenterol Hepatol.

[9] Burton AR, Pallett LJ, McCoy LE, Suveizdyte K, Amin OE, Swadling L (2018). Circulating and intrahepatic antiviral B cells are defective in hepatitis B. J Clin Invest.

[10] Salimzadeh L, Le Bert N, Dutertre CA, Gill US, Newell EW, Frey C (2018). PD-1 blockade partially recovers dysfunctional virus-specific B cells in chronic hepatitis B infection. J Clin Invest.

[11] Le Bert N, Salimzadeh L, Gill US, Dutertre C-A, Facchetti F, Tan A (2019). Comparative characterization of B cells specific for HBV nucleocapsid and envelope proteins in patients with chronic hepatitis B. J Hepatol.

[12] Zehn D, Utzschneider DT, Thimme R (2016). Immune-surveillance through exhausted effector T-cells. Curr Opin Virol.

[13] Maini MK, Burton AR (2019). Restoring, releasing or replacing adaptive immunity in chronic hepatitis B. Nat Rev Gastroenterol Hepatol.

[14] Vanwolleghem T, Groothuismink ZMA, Kreefft K, Hung M, Novikov N, Boonstra A (2020). Hepatitis B core-specific memory B cell responses associate with clinical parameters in patients with chronic HBV. J Hepatol.

[15] Sokal A, Chappert P, Barba-Spaeth G, Roeser A, Fourati S, Azzaoui I (2021). Maturation and persistence of the anti-SARS-CoV-2 memory B cell response. Cell.

[16] Jeffery-Smith A, Burton AR, Lens S, Rees-Spear C, Davies J, Patel M (2022). SARS-CoV-2-specific memory B cells can persist in the elderly who have lost detectable neutralizing antibodies. J Clin Invest.

[17] Herati RS, Knorr DA, Vella LA, Silva LV, Chilukuri L, Apostolidis SA (2022). PD-1 directed immunotherapy alters Tfh and humoral immune responses to seasonal influenza vaccine. Nat Immunol.

[18] Tan AT, Koh S, Goh W, Zhe HY, Gehring AJ, Lim SG (2010). A longitudinal analysis of innate and adaptive immune profile during hepatic flares in chronic hepatitis B. J Hepatol.

[19] Zhang J-W, Lai R-M, Wang L-F, Wang S-L, Xue H-X, Li C (2024). Varied immune responses of HBV-specific B cells in patients undergoing pegylated interferon-alpha treatment for chronic hepatitis B. J Hepatol.

[20] Milich DR, Mclachlan A (1986). The nucleocapsid of hepatitis B virus is both a T-cell-independent and a T-cell-dependent antigen. Science (80-).

[21] Bai L, Zhang X, Kozlowski M, Li W, Wu M, Liu J (2018). Extracellular Hepatitis B Virus RNAs Are Heterogeneous in Length and Circulate as Capsid-Antibody Complexes in Addition to Virions in Chronic Hepatitis B Patients. J Virol.

