# Supplementary material for: Tracking B cell immunity during perturbation of hepatitis B infection induced by treatment withdrawal
Source: Gut. Author manuscript; Available in PMC 2026 Sep 3. (PMC13422052; doi:10.1136/gutjnl-2024-333309)

Supplementary information

Title: Tracking B cell immunity during perturbation of hepatitis B infection outcome induced by treatment withdrawal

Index:

1. Methods.................. .... (page 1)
2. Supplementary Figures and Tables
   Suppl Table 1 .... (page 2)
   Suppl Table 2 .... (page 2)
   Suppl Table 3 .... (page 3)
   Suppl Figure 1 .... (page 4)
   Suppl Figure 2 .... (page 5)
   Suppl Figure 3 .... (page 6)
   Suppl Figure 4 .... (page 7)

Methods:

Virological serum and intrahepatic markers: Standard laboratory tests (upper limit normal for ALT= 40 IU/L) as well as determinations of HBeAg status and anti-HBe were performed by immunoassay using the Advia Centaur® System (Siemens, Erlangen, Germany). Serum HBV-DNA was determined by real-time PCR using the cobas® 6800 system (Roche Diagnostics, Manheim, Germany; LLQ <10 IU/ml). HBsAg was quantified using the ARCHITECT® HBsAg assay (Abbott Laboratories, Chicago, IL, USA; LLQ <0.13 IU/ml). HBcrAg levels were assessed by chemiluminescent enzyme immunoassay using LUMIPULSE® G1200 Analyzer (Fujirebio Europe, Gent, Belgium; LLQ <2.8 log U/ml) according to the manufacturer's instructions. 3.5 kb HBV-RNA (pregenomic RNA and precore mRNA) was assessed by real-time RT-PCR using specific primers and Taqman probe and the TaqMan® Fast Virus 1-Step Master Mix (Applied Biosystems, Thermo Fisher Scientific, Waltham, MA, USA). All patients underwent a liver biopsy prior to treatment withdrawal (baseline). This biopsy was divided into two parts, one for conventional histological analysis to rule out the presence of advanced fibrosis and the other part was stored in Allprotect Tissue Reagent (Qiagen, Hilden, Germany) at -80°C. Total intrahepatic HBV-DNA (iHBV-DNA) and cccDNA were determined by real-time PCR, as previously described[12][4].

Supplementary Figures and Tables

Suppl Table 1. List of mAbs used during the study and reference.

Marker	Concentration	Company	Cat number
CD3- BUV-805	1:100	BD Horizon	61289
CD19-BV-786	2:100	BD optibuild	740968
CD21-BV-421	2:100	BD Horizon	563815
CD27-BUV-395	1:100	BD Horizon	563815
CD4-BV-510	1:100	Biolegend	317444
IgD-PerCPCy5.5	2:100	Biolegend	348208
IgM-APCCy7	1:100	Biolegend	314520
CXCR5-FITC	3:100	BD Horizon	564624
PD1-BV605	1:100	Biolegend	329924
CD71-AF700	2:100	BD Pharmigen	563769
CD40-BV711	2:100	Biolegend	334334
CD40L-PECy7	2:100	Biolegend	310832
CD38-PE Dazzle	0.5:100	Biolegend	303538
DyLight650-PE	1:100	Gilead Sciences	n/a
DyLight550-APC	1:100	Gilead Sciences	n/a

Suppl Table 2. Patients characteristics at the time of NA discontinuation.

Variable	Study cohort<br>n=21 patients
Age (years)	57 (45-63)
Sex Male (n, %)	17 (81%)
Race Caucasian (n, %)	20 (95%)
HBV genotype<br>A<br>C<br>D<br>F	 2 (9.5%)<br>1 (4.8%)<br>17 (81%)<br>1 (4.8%)
Antiviral Therapy<br>- Tenofovir<br>- Entecavir	 16 (76%)<br>5 (24%)
Duration of antiviral therapy (years)	8 (7-13)
Transient Elastography (kPa)	4.7 (4-5.3)
Fibrosis stage<br>- F0-1<br>- F2	 19 (90.5%)<br>2 (9.5%)
Baseline ALT (IU/L)	23 (17-26)
Viral markers	
qHBsAg (IU/mL)	1405 (577-2780)
HBcrAg<br>- Positive<br>Log IU/mL	 8 (38%)<br>3 (2.8-3.25)
HBV-RNA<br>- Positive<br>- Copies/mL	 8 (38%)<br>591 (97-877)
iHBV-DNA (copies/cell)	0.393 (0.152-1)

cccDNA (copies/cell)	0.089 (0.039-0.255)

Suppl Table 3. Longitudinal qHBsAg (IU/mL) and anti-HBs (IU/mL) titers in patients achieving HBsAg loss (n=5).

	Marker	EOT	W12	W48	Late FU
Patient#1	qHBsAg	66	4	neg	neg
	Anti-HBs	neg	40	neg	21
Patient#2	qHBsAg	1755	32	neg	neg
	Anti-HBs	neg	neg	neg	1000
Patient#3	qHBsAg	329	345	neg	neg
	Anti-HBs	neg	neg	25	239
Patient#4	qHBsAg	74	4	neg	neg
	Anti-HBs	neg	neg	neg	161
Patient#5	qHBsAg	0.07	neg	neg	neg
	Anti-HBs	neg	135	25	NA

neg: negative or < LLOD. NA: not available.

Suppl Fig 1. Gating strategy of HBV-MBC and correlation with viral markers. (A) Gating strategy for HBs and HBc-specific memory B cells (MBC). (B) Correlation between HBs-MBC and qHBsAg. (C) Correlation between HBc-MBC and HBcrAg (only patients with HBcrAg+ positive are represented).

A

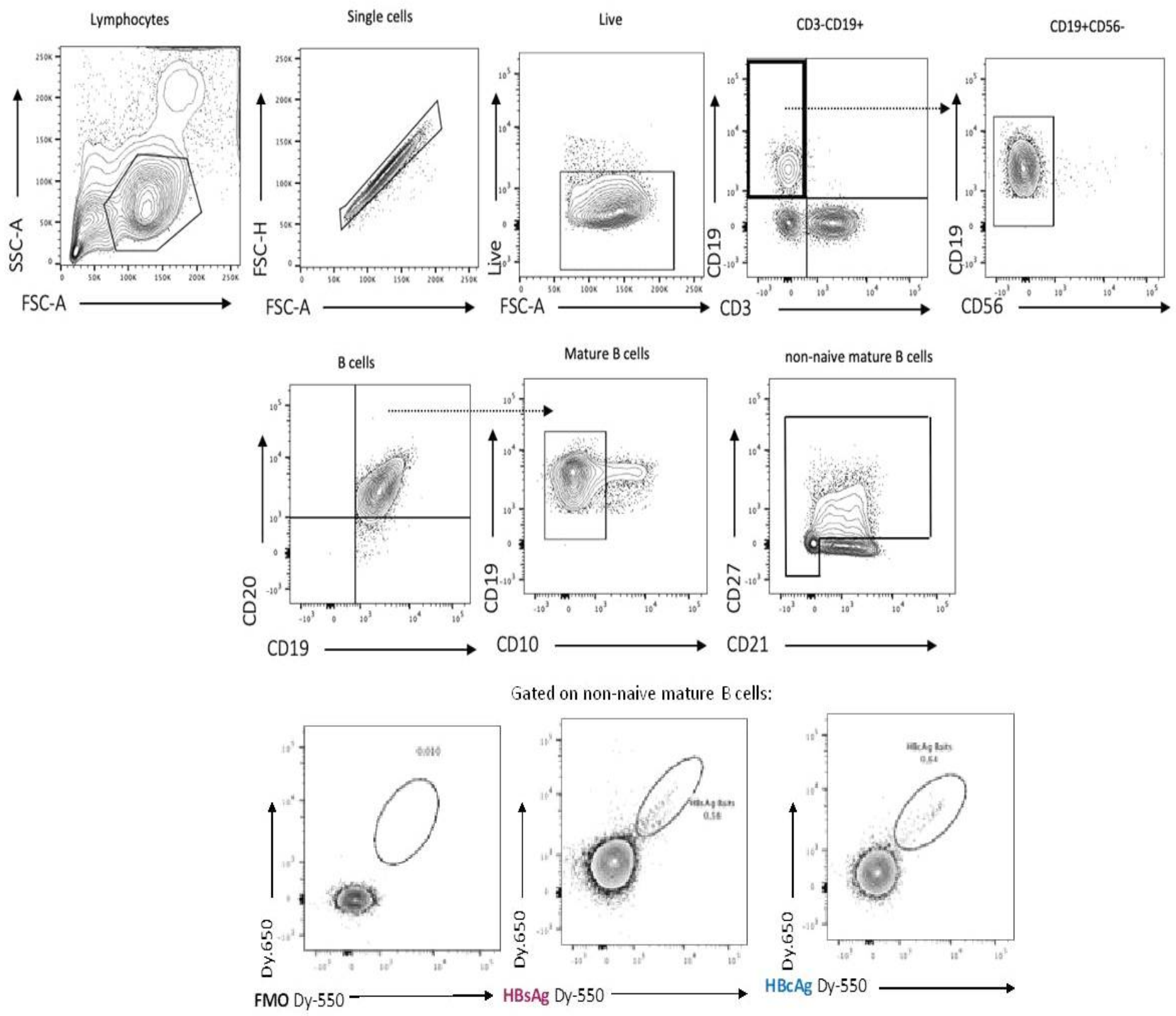

B

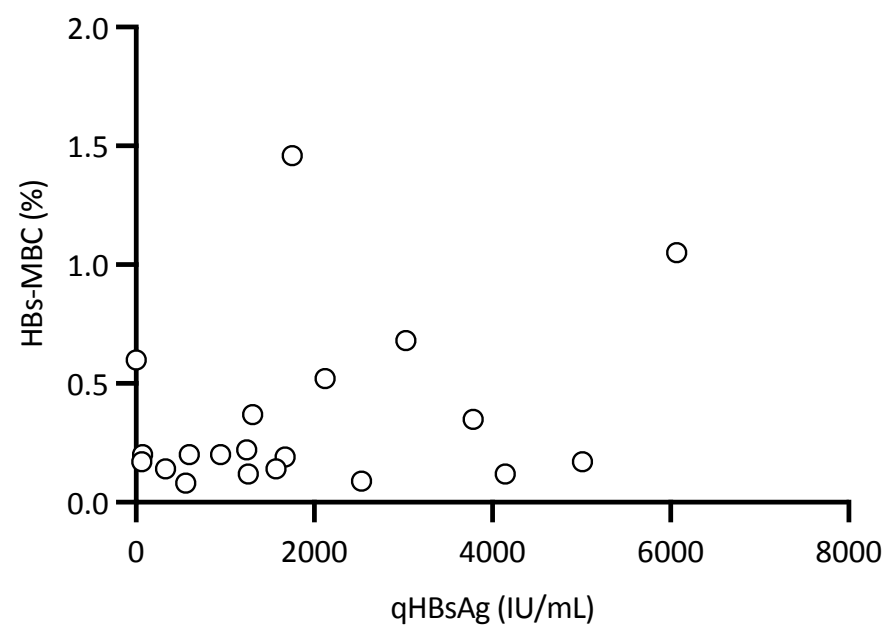

C

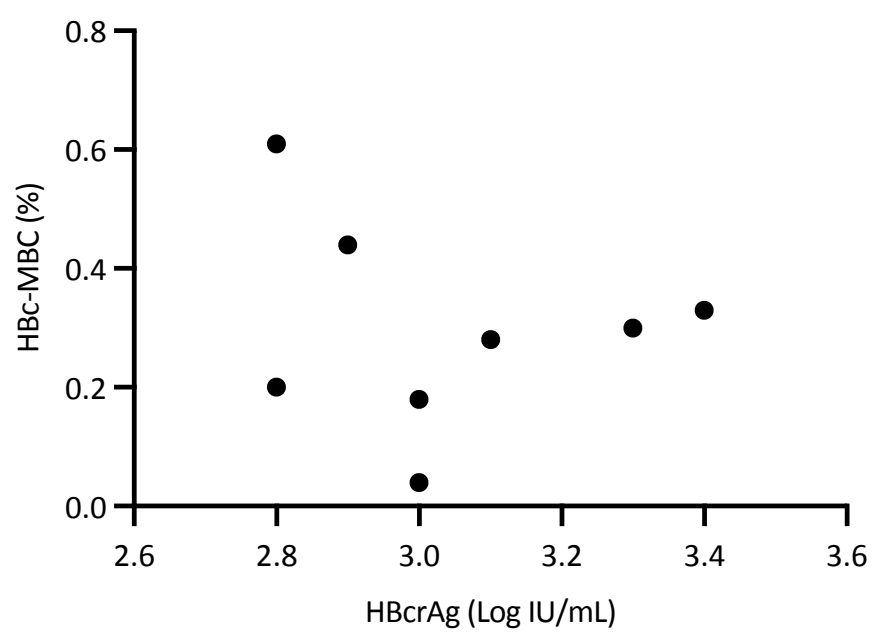

Suppl Fig 2. Patient outcome after NA discontinuation and predictors at EOT. (A) Outcomes after NA discontinuation. Bars represent the percentage of patients with HBsAg loss, virological control off-therapy, or re-treatment. (B) HBV-DNA, HBsAg, and ALT kinetics after NA discontinuation. For re-treated patients, the dotted line represents the evolution after treatment re-introduction. (C). HBsAg levels, cccDNA and iHBV-DNA in patients achieving HBsAg loss or not. (D) Distribution of MBC phenotype within HBs and HBc-MBC at EOT according to outcome (HBsAg loss or HBsAg+). Expression of phenotypic markers (CD40, CD71, CXCR5) on global B cells (E) and HBV-MBC (F) at EOT according to HBsAg outcome (dark blue dots: off-therapy HBsAg+ patients; light blue dots: re-treated HBsAg+ patients; Bars represent median). C-F Mann-Whitney. * p<0.05, ***p<0.001.

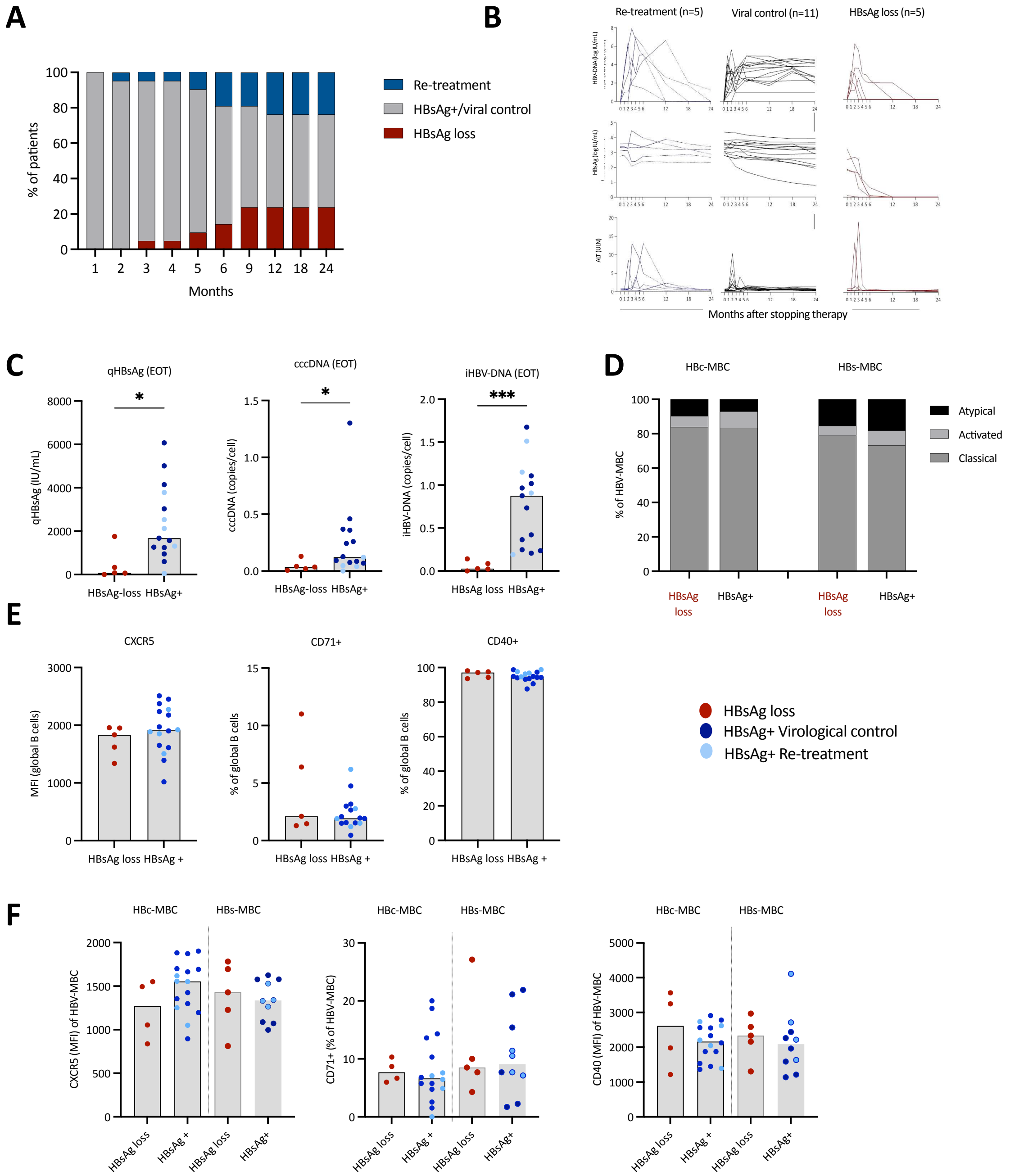

Suppl Fig 3. Longitudinal changes after NA discontinuation. (A) Change in frequencies of MBC subsets (classical, activated or atypical) within HBc-MBC between EOT and W12. (B) Changes in HBc-MBC according to HBcrAg increase. (C) HBs-MBC frequencies longitudinally in the group achieving HBsAg loss or not. Wilcoxon paired (A-B), ANOVA (C). * p<0.05, **p<0.01.

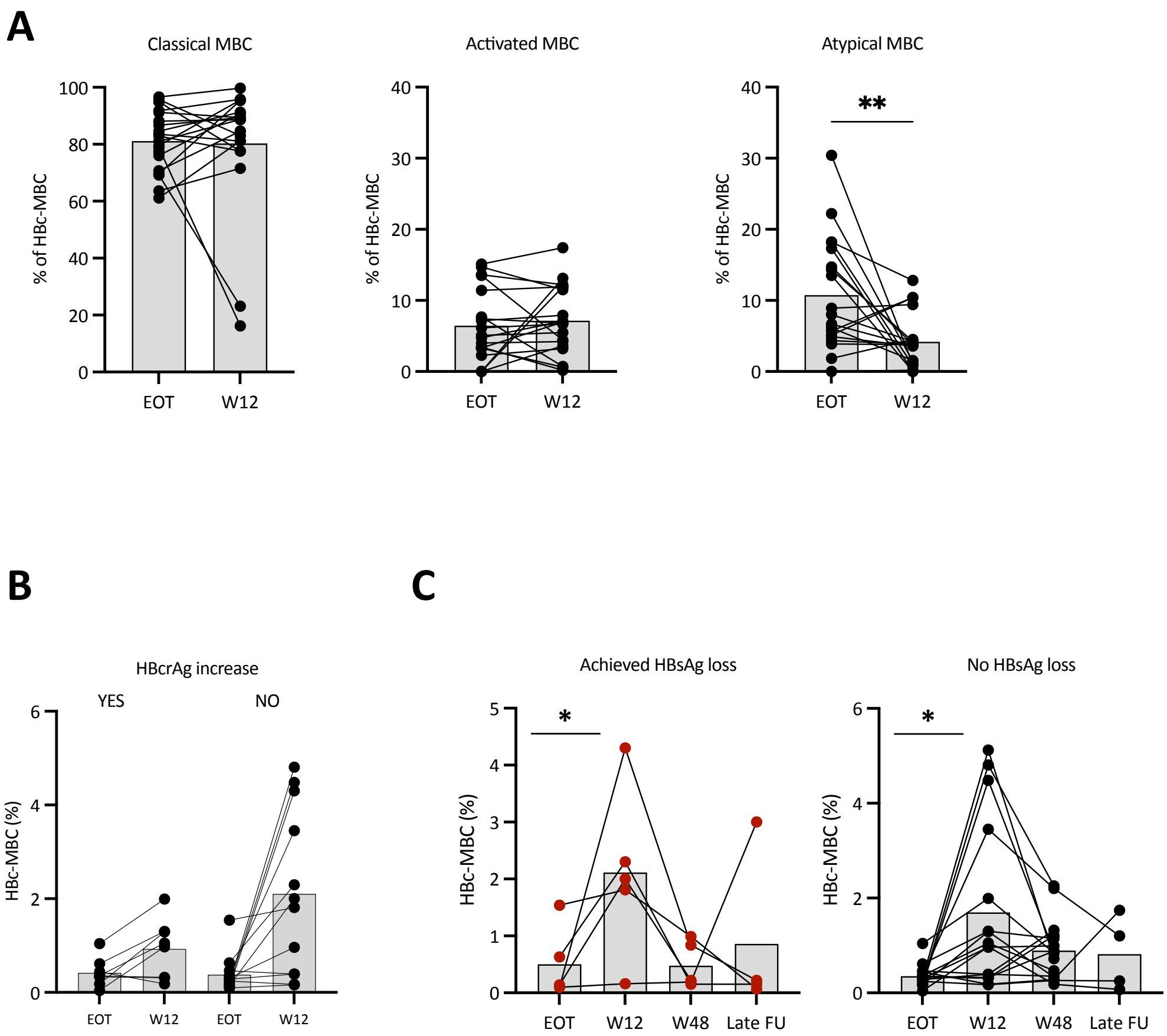

Suppl.Fig.4. B cell function and cTfh activation after HBsAg loss. (A) Frequencies of HBs-MBC at W48 according to clinical outcome (HBsAg loss or persistence). (B) Paired comparison of HBs and HBc antibody production measured by ELISpot and representative examples. (C) Correlation HBcAb and HBsAb by ELISpot at EOT (D) Individual HBcAb production by ELISpot according to HBsAg evolution (E) Frequencies of atypical MBC within HBs-MBC overtime. (F) Longitudinal CD38 expression on cTfh (G)) frequency of activated MBC according to qHBsAg outcome. (H) Frequencies of plasmablasts according to HBsAg status after NA discontinuation. Mann-Whitney (A), Wilcoxon (B) , Spearman correlation (C), ANOVA multiple comparisons (D-H).
EOT: end of treatment; W: week; FU: follow-up. ***p<0.001

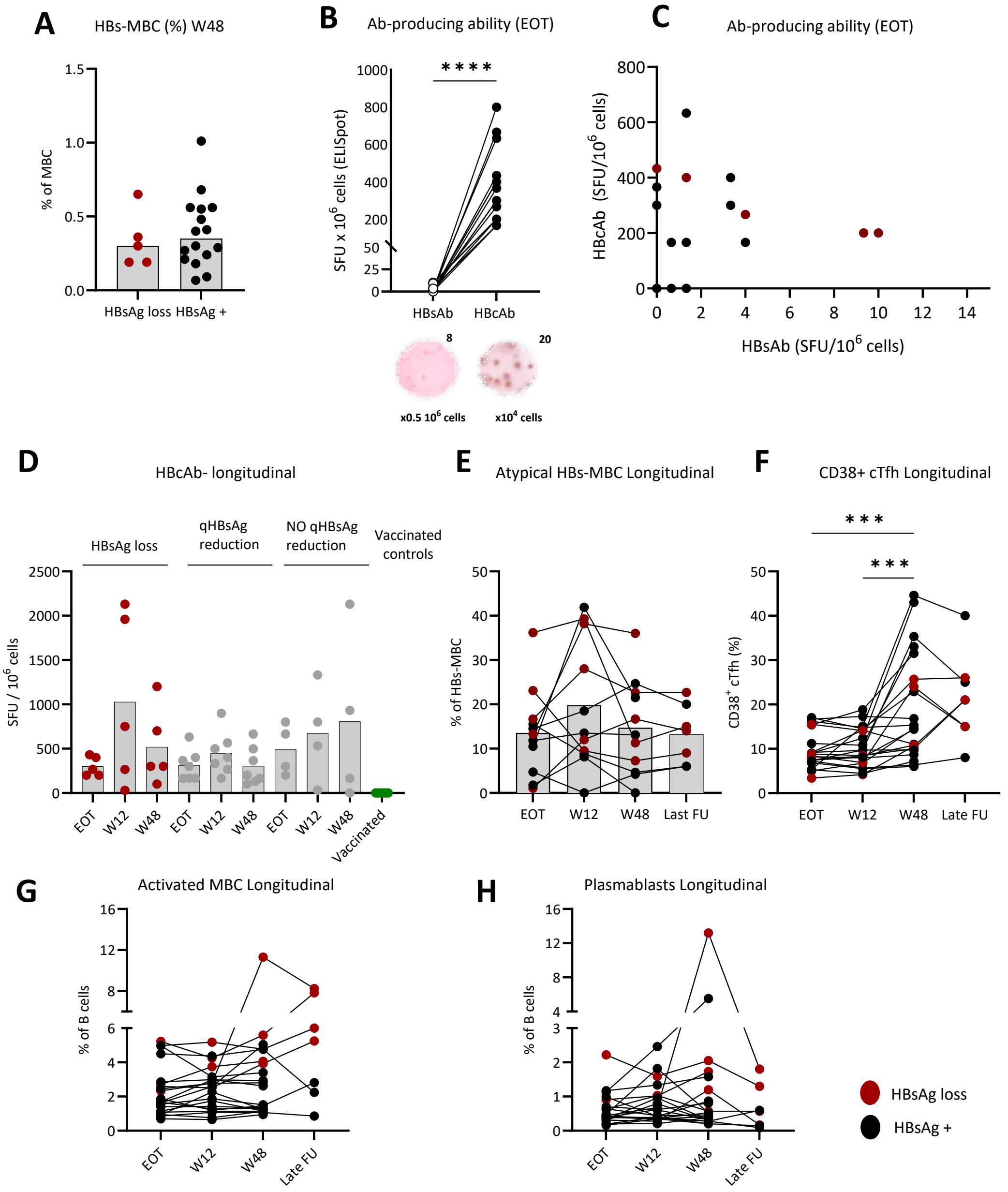

Supplement: Supplementary file 1 [file EMS216194-supplement-Supplementary_information.pdf]
