# Supplementary material for: Tracking B cell immunity during perturbation of hepatitis B infection induced by treatment withdrawal
Source: Gut. 2025 Dec 19;75(8):e333309. doi: 10.1136/gutjnl-2024-333309 (PMC13422052; doi:10.1136/gutjnl-2024-333309)
Supplement: online supplemental file 1 [file gutjnl-75-8-s001.docx]

**Supplementary information**

**Title: Tracking B cell immunity during perturbation of hepatitis B infection outcome induced by treatment withdrawal**

**Index**:

1. Methods................... ....................... (page 1)
2. Supplementary Figures and Tables

Suppl Table 1 ................................... (page 2)

Suppl Table 2 ................................... (page 2)

Suppl Table 3 ................................... (page 3)

Suppl Figure 1 ................................... (page 4)

Suppl Figure 2 ................................... (page 5)

Suppl Figure 3 ................................... (page 6)

Suppl Figure 4 ................................... (page 7)

**Methods:**

**Virological serum and intrahepatic markers:** Standard laboratory tests (upper limit normal for ALT= 40 IU/L) as well as determinations of HBeAg status and anti-HBe were performed by immunoassay using the Advia Centaur® System (Siemens, Erlangen, Germany). Serum HBV-DNA was determined by real-time PCR using the cobas® 6800 system (Roche Diagnostics, Manheim, Germany; LLQ <10 IU/ml). HBsAg was quantified using the ARCHITECT® HBsAg assay (Abbott Laboratories, Chicago, IL, USA; LLQ <0.13 IU/ml). HBcrAg levels were assessed by chemiluminescent enzyme immunoassay using LUMIPULSE® G1200 Analyzer (Fujirebio Europe, Gent, Belgium; LLQ <2.8 log U/ml) according to the manufacturer’s instructions. 3.5 kb HBV-RNA (pregenomic RNA and precore mRNA) was assessed by real-time RT-PCR using specific primers and Taqman probe and the TaqMan® Fast Virus 1-Step Master Mix (Applied Biosystems, Thermo Fisher Scientific, Waltham, MA, USA). All patients underwent a liver biopsy prior to treatment withdrawal (baseline). This biopsy was divided into two parts, one for conventional histological analysis to rule out the presence of advanced fibrosis and the other part was stored in Allprotect Tissue Reagent (Qiagen, Hilden, Germany) at -80°C. Total intrahepatic HBV-DNA (iHBV-DNA) and cccDNA were determined by real-time PCR, as previously described[12][4].

**Supplementary Figures and Tables**

**Suppl Table 1**. List of mAbs used during the study and reference.

| Marker | Concentration | Company | Cat number |
| --- | --- | --- | --- |
| CD3- BUV-805 | 1:100 | BD Horizon | 61289 |
| CD19-BV-786 | 2:100 | BD optibuild | 740968 |
| CD21-BV-421 | 2:100 | BD Horizon | 563815 |
| CD27-BUV-395 | 1:100 | BD Horizon | 563815 |
| CD4-BV-510 | 1:100 | Biolegend | 317444 |
| IgD-PerCPCy5.5 | 2:100 | Biolegend | 348208 |
| IgM-APCCy7 | 1:100 | Biolegend | 314520 |
| CXCR5-FITC | 3:100 | BD Horizon | 564624 |
| PD1-BV605 | 1:100 | Biolegend | 329924 |
| CD71-AF700 | 2:100 | BD Pharmigen | 563769 |
| CD40-BV711 | 2:100 | Biolegend | 334334 |
| CD40L-PECy7 | 2:100 | Biolegend | 310832 |
| CD38-PE Dazzle | 0.5:100 | Biolegend | 303538 |
| DyLight650-PE | 1:100 | Gilead Sciences | n/a |
| DyLight550-APC | 1:100 | Gilead Sciences | n/a |

**Suppl Table 2**. Patients characteristics at the time of NA discontinuation.

| Variable | Study cohort  n=21 patients |
| --- | --- |
| Age (years) | 57 (45-63) |
| Sex Male (n, %) | 17 (81%) |
| Race Caucasian (n, %) | 20 (95%) |
| HBV genotype  A  C  D  F | 2 (9.5%)  1 (4.8%)  17 (81%)  1 (4.8%) |
| Antiviral Therapy   - Tenofovir - Entecavir | 16 (76%)  5 (24%) |
| Duration of antiviral therapy (years) | 8 (7-13) |
| Transient Elastography (kPa) | 4.7 (4-5.3) |
| Fibrosis stage   - F0-1 - F2 | 19 (90.5%)  2 (9.5%) |
| Baseline ALT (IU/L) | 23 (17-26) |
| Viral markers | |
| qHBsAg (IU/mL) | 1405 (577-2780) |
| HBcrAg   - Positive   Log IU/mL | 8 (38%)  3 (2.8-3.25) |
| HBV-RNA   - Positive - Copies/mL | 8 (38%)  591 (97-877) |
| iHBV-DNA (copies/cell) | 0.393 (0.152-1) |
| cccDNA (copies/cell) | 0.089 (0.039-0.255) |

**Suppl Table 3**. Longitudinal qHBsAg (IU/mL) and anti-HBs (IU/mL) titers in patients achieving HBsAg loss (n=5).

|  | Marker | EOT | W12 | W48 | Late FU |
| --- | --- | --- | --- | --- | --- |
| Patient#1 | qHBsAg | 66 | 4 | neg | neg |
|  | Anti-HBs | neg | 40 | neg | 21 |
| Patient#2 | qHBsAg | 1755 | 32 | neg | neg |
|  | Anti-HBs | neg | neg | neg | 1000 |
| Patient#3 | qHBsAg | 329 | 345 | neg | neg |
|  | Anti-HBs | neg | neg | 25 | 239 |
| Patient#4 | qHBsAg | 74 | 4 | neg | neg |
|  | Anti-HBs | neg | neg | neg | 161 |
| Patient#5 | qHBsAg | 0.07 | neg | neg | neg |
|  | Anti-HBs | neg | 135 | 25 | NA |

neg: negative or < LLOD. NA: not available.
