# Supplementary material for: Tracking B cell immunity during perturbation of hepatitis B infection induced by treatment withdrawal
Source: Gut. 2025 Dec 19;75(8):e333309. doi: 10.1136/gutjnl-2024-333309 (PMC13422052; doi:10.1136/gutjnl-2024-333309)
Supplement: online supplemental file 2 [file gutjnl-75-8-s002.pptx]

## Slide 1
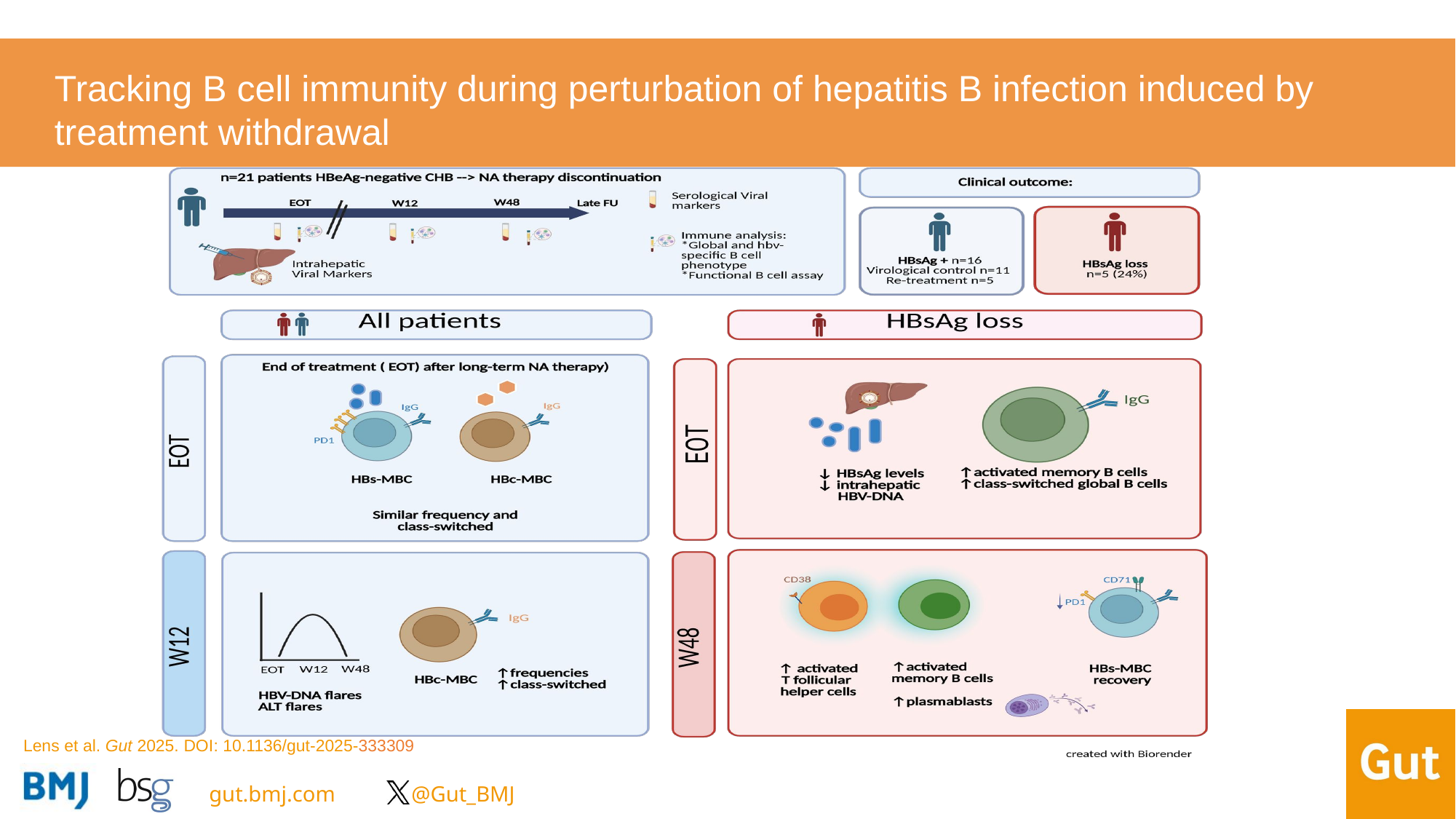

Tracking B cell immunity during perturbation of hepatitis B infection induced by treatment withdrawal
Lens et al. Gut 2025. DOI: 10.1136/gut-2025-333309
gut.bmj.com
@Gut_BMJ
